# The emerging role of FTY720 (Fingolimod) in cancer treatment

**DOI:** 10.18632/oncotarget.7145

**Published:** 2016-02-02

**Authors:** Christopher White, Heba Alshaker, Colin Cooper, Matthias Winkler, Dmitri Pchejetski

**Affiliations:** ^1^ School of Medicine, Imperial College London, London, UK; ^2^ Department of Pharmacology and Biomedical Sciences, Faculty of Pharmacy and Medical Sciences, University of Petra, Amman, Jordan; ^3^ School of Medicine, University of East Anglia, Norwich, UK; ^4^ Department of Surgery and Cancer, Imperial College London, London, UK

**Keywords:** sphingolipid, sphingosine kinase, fingolimod, FTY720, cancer

## Abstract

FTY720 (Fingolimod) is a clinically approved immunomodulating therapy for multiple sclerosis that sequesters T-cells to lymph nodes through functional antagonism of sphingosine-1-phosphate 1 receptor. FTY720 also demonstrates a proven efficacy in multiple *in vitro* and *in vivo* cancer models, suggesting a potential therapeutic role in cancer patients. A potential anticancer mechanism of FTY720 is through the inhibition of sphingosine kinase 1, a proto-oncogene with *in vitro* and clinical cancer association. In addition, FTY720's anticancer properties may be attributable to actions on several other molecular targets. This study focuses on reviewing the emerging evidence regarding the anticancer properties and molecular targets of FTY720. While the clinical transition of FTY720 is currently limited by its immune suppression effects, studies aiming at FTY720 delivery and release together with identifying its key synergetic combinations and relevant patient subsets may lead to its rapid introduction into the clinic.

## TARGETING SPHINGOLIPID SIGNALLING FOR CANCER TREATMENT

### Introduction to sphingolipid metabolism

**Figure 1:**
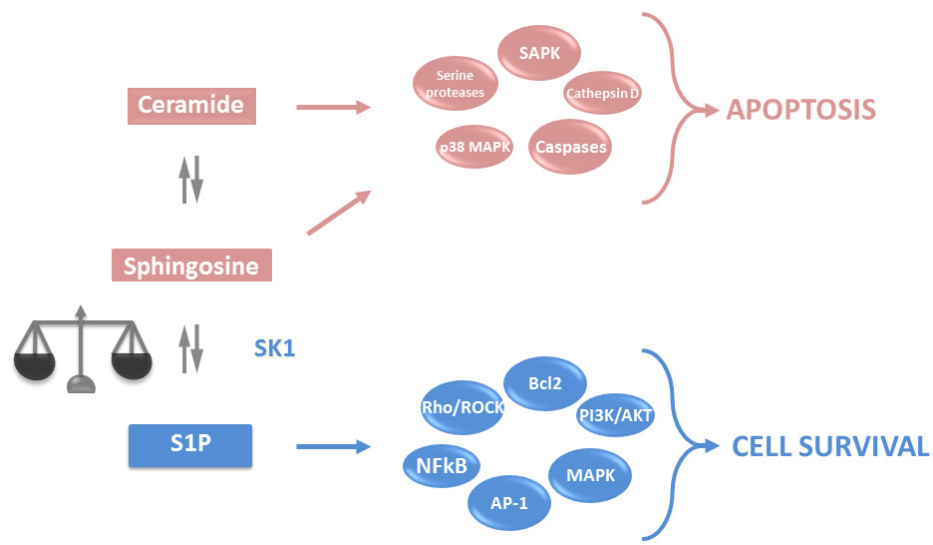
Sphingolipid rheostat Ceramide and sphingosine are intracellular lipid second messengers, which induce activation of apoptotic pathways. In turn, SK1 can phosphorylate sphingosine to yield S1P, a lipid second messenger that activates anti-apoptotic pathways and antagonises the effects of ceramide and sphingosine. The intracellular balance between ceramide, sphingosine and S1P determines the cell fate. PKC - protein kinase C; MAPK - mitogen-activated protein kinases; JNK - c-Jun N-terminal kinases; SAPK - stress activated protein kinase; NFkB - nuclear factor kappa B; PLC - phospholipase C; Bcl2 - B cell lymphoma 2, AP-1 - activator protein 1 (reviewed in [248] and [249]).

Sphingolipids are one of the major components of eukaryotic cell plasma membranes. Aside from their structural role, they have attracted attention as potent second messengers regulating programmed cell death. Cleavage of a pro-apoptotic sphingolipid ceramide yields pro-apoptotic sphingosine that is phosphorylated by sphingosine kinases (SKs) to anti-apoptotic sphingosine-1-phosphate (S1P) (Figure 1). The dynamic balance between S1P and sphingosine/ceramide signalling is referred to as the “sphingolipid rheostat” and determines whether a cell undergoes apoptosis (reviewed in [1-3]). S1P can be dephosphorylated or degraded [4] (Figure 1), and the balance of production and degradation of S1P is tightly regulated (reviewed in [5]). Importantly, the enzymes of the rheostat do not just function by directly changing the balance of metabolites, but also by the roles these metabolites have in a myriad of signaling pathways with production, localisation, secretion and signaling of these metabolites having profound effect on tumor outcomes [6].

**Figure 2:**
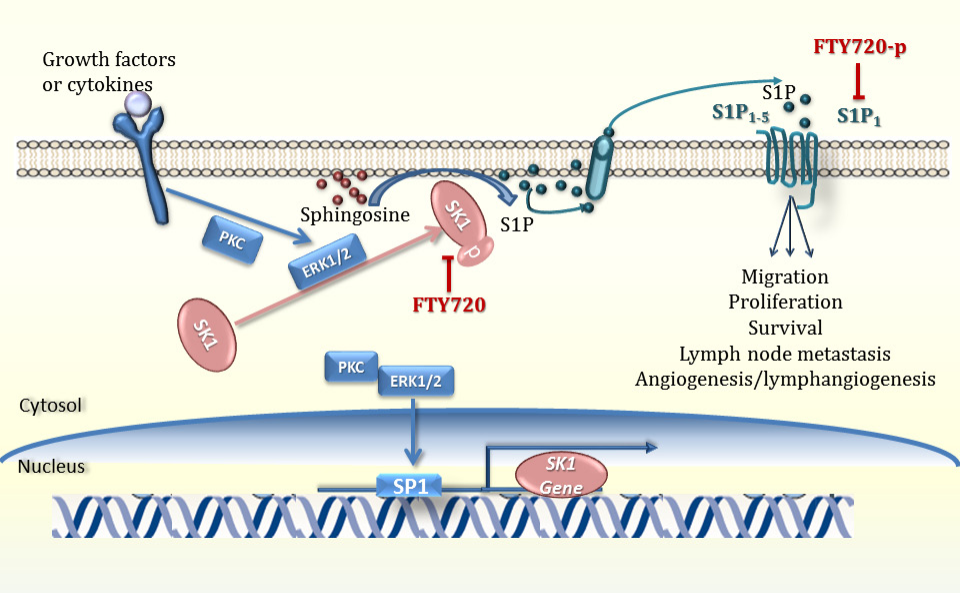
SK1/S1P signalling pathway. Tumour necrosis factor alfa, growth factors and cytokines activate receptor tyrosine kinases, G-protein coupled receptors, toll like receptors, which induce phosphorylation of sphingosine kinase 1 (SK1, often through ERK1/2 and PKC), its translocation to plasma membrane and generation of sphingosine-1-phosphate (S1P) from sphingosine (reviewed in [250]). S1P can then be exported outside of the cell where it acts in a paracrine or autocrine manner and activates 5 specific S1P receptors (S1P1-5). Upon coupling with S1P, these receptors can activate downstream signalling pathways leading to cell proliferation, migration and gene expression. PKC - protein kinase C; (reviewed in [248] and [249]).

Two SK genes expressed in humans, SK1 and SK2, display different catalytic properties [7] suggesting distinct physiological roles [8-10]. SKs possess an intrinsic catalytic activity [11] which is rapidly accelerated upon phosphorylation, [12] inducing its translocation to the plasma membrane [11]. SKs may have extracellular effects (reviewed in [13]). SK1 or SK2 single knockout in mice does not affect development and reproduction, whereas simultaneous knockout results in S1P deficiency and embryonic lethality [14]. SK1 is a proto-oncogene and is regulated through multiple mechanisms. Upon stimulation, SK1, located predominately in the cytosol, translocates to the plasma membrane and enhances S1P secretion and proliferative signalling [15] (Figure 2). Through binding cell surface S1P G-protein coupled receptors (S1PRs1-5), S1P initiates autocrine and paracrine signalling cascades that induce cell migration, angiogenesis and differentiation (reviewed in [16], (Figure 2). Diverse outcomes of S1P signalling depend on the cell type and the expression of G proteins and S1PRs [17]. Acting as an intracellular second messenger S1P enhances proliferation and suppresses apoptosis (reviewed in [16]. Internalised upon ligand binding, S1PRs can then either resensitise or degrade [18] depending on ubiquitination status. S1P binding through mono-ubiquitination leads to resensitisation, whilst other agonists (e.g. FTY720) lead to degradation through poly-ubiquitination [19]. S1P has several non-receptor intracellular actions (reviewed in [20]) including binding histone deacetylases HDAC1 and HDAC2 and regulating gene expression epigenetically [21], and forming complexes with tumour necrosis factor (TNF) receptor-associated factor 2 (TRAF2) leading to increased nuclear factor kappa B (NF-kB) signalling [22].

In healthy cells, ceramide and sphingosine play a crucial role in physiological apoptotic machinery while S1P signalling leads to cell proliferation, migration, angiogenesis, inflammatory response and lymphocyte trafficking. In cancer cells, dysregulation of enzymes involved in sphingolipid metabolism to escape cell death leads to increased S1P signalling, often through aberrant expression of ceramide degrading enzymes, sphingosine kinases or S1PRs (reviewed in [23]). While this provides rationale for therapeutic targeting of these pathways, their important physiological role in other tissues (such as heart or liver) urges for extreme caution. In particular, targeting S1P may lead to lymphocyte retention in lymph nodes and subsequent lymphopenia, which would be an undesirable side effect, especially in cancer patients.

### Sphingosine kinase 1 as a potential therapy target for cancer treatment

#### Clinical and *in vitro* association

Compelling evidence suggests that SK1 activation contributes to cancer progression. High SK1 expression has been shown in several human cancers including brain, breast, colon, lung, ovary, stomach, uterus, kidney, rectum and small intestine [24-27]. Expression of high levels of S1P receptors, S1P1 and S1P3, SK1, and extracellular signal-regulated kinase-1/2 are associated with development of tamoxifen resistance in estrogen receptor-positive breast cancer patients [28]. This is the first study to demonstrate the association of survival rates and disease recurrence with combined S1P1/S1P3 and SK1 protein expression indicating a complex relationship between S1P receptor and SK1 expression and outcomes. This may indicate the significance of the autocrine activation of this pathway in breast cancer cells and suggests that disruption of this pathway may provide a target for treatment of tamoxifen-resistant breast cancer [28].

High levels of SK1 expression/activity are associated with poor prognosis, decreased survival rate [25] and histologic grade [29] in glioma; poor prognosis in breast cancer patients [30]; prostate cancer progression (Prostate specific antigen (PSA), tumour volumes and Gleason score) and disease recurrence (positive margins and surgical failure) [31]; shorter survival time in gastric cancer patients [32]; poor survival and tumour progression in non-small cell lung cancer [33]; TNM status, tumour differentiation and shorter overall survival time in salivary gland cancer [34]; and advanced tumour stage, nodal involvement, recurrence, shorter patient survival time and loss of p21 expression in head and neck cancer [35, 36].

These associations have complex pathophysiological mechanisms. A hallmark study showed that enforced expression of SK1 is sufficient for malignant transformation of NIH-3T3 fibroblasts leading to serum independence and tumour formation [37]. The expression of SK1a and SK1b in androgen-independent LNCaP-AI prostate cancer cells is upregulated compared with androgen-sensitive LNCaP prostate cancer cells, suggesting that androgen escape might be associated with increased transcriptional up-regulation of SK1a/b [38]. Indeed, long-term androgen deprivation raises basal SK1 levels in prostate cancer cells, although the exact mechanism is not known [39]. This is confirmed in androgen-independent prostate cancer cells derived from patients' brain and bone metastases which have ~10-fold higher SK1 activity than androgen dependent prostate cancer cells derived from lymph nodes [40].

#### Protection against apoptosis

Many studies have shown that one of the major functions of SK1 is to provide cancer cells protection from apoptosis. Thus, targeting SK1 was quickly proposed as a potential therapeutic approach for cancer treatment. Indeed, many cancer cell lines are sensitive to treatment with either siRNAs to SK1 or pharmacological inhibitors of this enzyme [27, 40, 41] independently of p53 mutation [40] or Bcl-2 status [42]. SK1 is upregulated in response to several anticancer treatments [40, 43, 44] leading to resistance of cancer cells to these therapies. Apoptosis-induced SK1 expression and subsequent release of S1P signals to tumour-associated macrophages and may therefore promote an inflammatory tumour microenvironment [45]. SK1 expression can protect the cells against apoptosis induced by TNF-α and Fas ligand [46, 47], and can mediate survival under stress conditions such as starvation [37, 48].

#### Inflammatory response

In addition to blocking cancer cell death, it has been proposed that SK1 promotes pro-inflammatory cytokine release [49]. Extracellular S1P induced COX2 overexpression and PGE2 production in L929 fibrosarcoma and A549 lung adenocarcinoma cells [50]. S1P secreted from apoptotic tumour cells could induce macrophage polarisation [51] and stimulated chemotaxis of primary monocytes and macrophages, whereas S1P antibody abrogated macrophage invasion to ischemic areas [52]. Tumour associated macrophages (TAMs) are strongly associated with a poor prognostic outcome in cancer patients and induce TNFα-dependent activation of JNK and NF-κB in adjacent tumour cells to promote their growth, motility and invasion [53, 54]. TAMs secrete promigratory cytokines/chemokines, including those released in response to activation of the SK1/S1P pathway [50, 55]. The SK1/S1P pathway is involved in inflammatory responses to cytokines such as TNFα and interleukin (IL-1) [56]. TNFα, via a TRAF2-dependent mechanism, activates SK1 leading to activation of the pro-survival and pro-inflammatory pathways mediated by AKT [57, 58] and NF-κB [58] through ubiquitination of receptor interacting protein 1 and stimulation of IκB kinase [22]. However, in both murine macrophages lacking both *SK1* and *SK2* and WT macrophages, TNFα and LPS induced similar inflammatory responses and activated the NFκB pathway to a similar extent, possibly suggesting that intracellular S1P is not necessary for the activation of this critical inflammatory signaling pathway [59].

#### Migration

Activation of SK1 downstream of several chemotactic receptors (e.g. lysophosphatidic acid (LPA1). epidermal growth factor or platelet-derived growth factor) [60, 61] enhances metastatic potential of cancer cells [62, 63] and cancer cell migration [64-68]. In many instances SK1-induced cell migration is mediated by S1P secretion and coupling to S1P receptors [69]. S1P1, S1P3 and S1P4 receptors mediate promigratory responses [70-72][50] through activation of Rac signalling, actin polymerization and lamellipodia formation. S1P2 (except in fibroblasts [73]) and S1P5 mediate cessation of migration through stimulation of Rho and Rac leading to stress fiber formation [74-76], suggesting the effect of S1P depends on differential expression of S1PRs in a specific cell type. In U373 glioblastoma cells SK1/S1P-induced cancer cell migration was linked with expression of plasminogen activator inhibitor-1 (PAI-1) and urokinase receptor (uPAR) [77]. Induction of cancer cell migration may also occur through intracellular non-receptor mechanisms, for example in hepatocyte growth factor-induced migration of endothelial cells [78]. The formation of a signalling complex between SK1, S1P1 and the cytoskeletal protein filamin A that localises to membrane ruffes of migrating cells to promote cell movement has been reported [66].

#### Neovascularisation

SK1/S1P signalling enhances tumour neovascularisation [79]. S1P secreted from tumour tissue can act as a chemoattractant for various cells including vascular endothelial cells [79]. S1P promotes endothelial-cell growth and interacts with vascular endothelial growth factor VEGF signalling [80]. VEGF stimulated S1P production mediated activation of RAS and MAPKs in T24 bladder tumour cells [81]. S1P1 expression is strongly induced in tumour vessels and specific knockdown of S1P1 significantly abrogates angiogenesis in murine models [82]. Secreted S1P initiated endothelial cell sprouting in 3-dimensional collagen matrices [83]. Antibodies to S1P have antitumour potential [63] through inhibition of cell proliferation, release of proangiogenic cytokines (e.g. VEGF, IL-8 and IL-6) and blocking S1P-related angiogenesis [63].

#### Chemoresistance

SK1 plays a role in chemoresistance and SK1 inhibition is proposed to correlate with chemotherapy efficiency [40]. SK1 overexpression inhibits chemotherapy-induced apoptosis: anthracyclines in MCF-7 breast cancer cells [47]; doxorubicin and etoposide in HL-60 acute myeloid cells [41]; camptothecin and docetaxel in PC3 and LNCaP prostate cancer cells [40]; and MDR-associated chemoresistance in an acute myeloid leukemia (AML) model [41]. In prostate cancer cell lines and animal models indirect SK1 inhibition was a valid chemotherapeutic strategy [84]. Modulation of SK or S1P lyase has been suggested to contribute to altered sensitivity to cisplatin [85]. *In vitro* and *in vivo* models of prostate cancer demonstrated that the SK1/S1P pathway has the potential to synergise with the effects of camptothecin chemotherapy [86], docetaxel chemotherapy [87] and radiotherapy [88]. SK1 inhibition restored endocrine response in breast cancer cells [89], and decreased colony formation [90], cell motility and chemotaxis [49, 91]. Pharmacological inhibition of SK1 results in resensitisation to anticancer therapies [41, 92, 93], notably through targeting SK1 to the ubiquitin-proteasomal degradation pathway and lowering SK1a/b levels below a threshold required for survival [38].

### Therapeutic potential of sphingosine kinase 1 inhibition

SK1 is a potential target in cancer therapy. Dimethylsphingosine (DMS), a non-selective SK inhibitor [94] and its methylated derivative N, N-dimethylsphingosine (DMS) induce apoptosis in numerous cancer cells [94, 95], reviewed in [96]), inhibit *in vivo* growth of lung and gastric carcinoma tumours in athymic mice [97], decrease lung metastasis of melanoma cells in syngeneic mice [98], and induce apoptosis and sensitise LNCaP cells to gamma-irradiation-induced apoptosis [99]. Lacking specificity, DMS inhibits protein kinase C, phospholipase A2, and phospholipase D [100].

F-12509A and B-5354c are SK inhibitors with greater specificity [101]. F-12509A induces cancer cell apoptosis in imatinib-resistant cells [102], and in HL-60, HL-60/Doxo and HL-60/VP16 cells leading to nuclear fragmentation, caspase-3 cleavage, downregulation of XIAP, cytochrome C and SMAC/Diablo release [41]. B-5354c induces apoptosis in LNCaP and PC-3 prostate cancers which is reversed by upregulation of SK1 [86].

2-(p-hydroxyanilo)-4-(p-chlorophenyl)thiazole (SKI-II), a SK-selective inhibitor has anti-cancer effects. SKI-II is cytotoxic to T24 bladder carcinoma cells, and MCF-7 and MCF-7/VP breast cancer cells [27]. SKI-II induces apoptosis in LNCaP and PC-3 human prostate cancer cells, irrespective of their p53 status [40]. Upon intraperitoneal administration of SKI-II, tumour size decreases and tumour growth is inhibited by 50-80% [103]. SKI-II abrogates androgen receptor signalling via an oxidative stress-induced, p53-independent mechanism in prostate cancer cells [104].

Selective SK1 inhibitor (SK1-I) ([(2-hydroxy-1-naphthyl)methylene]-3-(2-napythyl)-1H-pyrazole-5-carbohydrazide) induces apoptosis of leukaemia cells but not normal bone marrow derived cells [105].

Further SK1-specific inhibitors have been developed through modifications of sphingosine [106], and amidine-based subtype-selective SK1 inhibitors. These inhibitors induce reduction of endogenous S1P levels in human leukemia cells at nanomolar concentrations [107]. (S)-FTY720 vinylphosphonate [108] and sphingo-guanidines (LCL146 and LCL351) [109] induce SK1 inhibition in breast and prostate cancer cells and decrease migration rate of human prostate DU145 cells. New SK inhibitors optimised for selectivity and activity include SK1-178, which is active *in vitro* and *in vivo* and may help discern the role the SK1 and SK2 in disease development and progression [110].

L-threo-dihydrosphingosine (safingol) has sphingosine kinase-inhibiting properties [111]. A Phase I clinical trial of safingol, in combination with cisplatin in 43 cancer patients, reported safe use, reduction in S1P in plasma, significant regression of liver and lung metastases in one adrenal cortical cancer patient, and prolonged stable disease in another patient [112].

More recently, SK1 inhibitors with sub-micromolar potency have been more thoroughly characterized.  In several studies, these more selective SK1 inhibitors did not demonstrate cytotoxic effects. For example, PF-543, with a K(i) of 3.6nM and an IC50 of 2nM for SK1, had no effect on proliferation and survival of various cancer cell lines including head and neck carcinoma cells [113]. Through use of sub-micromolar amidine-based SK1 inhibitors, a lack of correlation between SK1 inhibition with changes in cell survival in U937, Jurkat T and SKOV3 cells was demonstrated [114]. Potent and specific SK1/2 inhibitors completely inhibited intracellular S1P production in human cells and attenuated vascular permeability in mice, but did not lead to reduced tumor cell growth *in vitro* or *in vivo* [115]. While the cytotoxic effects demonstrated by older less specific SK1 inhibitors may be explained by their off-target effects rather than by their action on SK1, there is significant evidence showing anticancer cytotoxic effects of SK1 siRNAs [116-119]. Conversely, one recent paper showed that siRNA targeting SK1 in a large panel of cell lines failed to demonstrate any statistically significant effects on cell viability [115].

## FTY720 AS A NEW MOLECULAR THERAPY FOR CANCER TREATMENT

### FTY720

**Figure 3:**
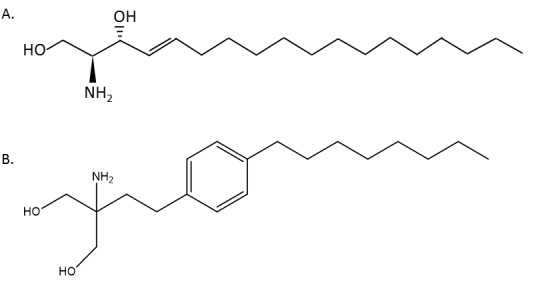
Structures of sphingosine (A) and FTY720 (B)

FTY720 (Fingolimod, Gilenya) (Figure 3) is a structural analogue of sphingosine developed from the fungal metabolite myriocin [120]. A phenylene moiety in FTY720's side chain confers potent immunosuppressive activity [121]. FTY720 is phosphorylated by SK2 to form FTY720-phosphate (FTY720-p), but is not phosphorylated, or phosphorylated with low efficiency, by SK1 [88, 108, 122-128]. FTY720-p is an agonist of S1Prs 1, 3, 4 and 5 and same time a functional antagonist of S1PR1 receptor [124, 129]. Through internalization and degradation of lymphocytes' S1PR1 receptor, FTY720 inhibits lymphocytes' egress from secondary lymphoid tissues and thymus and induces lymphopaenia [71, 130-134]. In multiple sclerosis FTY720 acts upon naïve and central memory T-cells without affecting peripheral effector memory cells [135]. The U.S. Food and Drug Administration (FDA) have approved FTY720 as a first-line treatment in relapsing forms of multiple sclerosis [136]. Owing to its cardio-protective effects FTY720 is a candidate for heart failure and arrhythmia treatment [137-140]. FTY720 has failed phase III clinical trials as an immunosuppressant for use in kidney transplantation [141, 142].

### Anti-cancer properties of FTY720

FTY720 demonstrates anti-cancer properties and may have potential in cancer treatment. *In vitro* and *in vivo* studies demonstrate the growth arrest and apoptosis-inducing ability of FTY720 in diverse normal and cancer cells including: lymphocytes [143, 144], microglia [145], prostate cancer [88, 108, 146-149], breast cancer [108, 122, 150, 151], several forms of leukaemia and lymphoma [152-159], lung cancer [160-162], liver cancer [163-166], pancreatic cancer [167, 168], bladder cancer [169], renal cancer [170, 171]; glioma [172], gastric cancer [173], colon cancer [151, 174] and ovarian cancer [175].

FTY720 is also a therapy sensitiser. Treatment of colorectal cancer cells with FTY720 shows an additive effect with 5-fluorouracil, SN-38, and oxaliplatin [176], and results in resensitization to cetuximab both *in vitro* and *in vivo* with inhibition of tumour growth, interference with signal transduction, induction of cancer cells apoptosis and prolongation of mice survival [177]. FTY720 significantly augments treatment efficacy and overall survival of mice receiving allogeneic adoptive cell transfer [178].

FTY720 inhibits metastasis in a mouse model of melanoma [179] and glioblastoma cell lines [180], and is able to inhibit microvessel formation and reduce expression of the angiogenesis promoting factor VEGF in androgen independent prostate tumour xenograft in nude mice [146]. FTY720 has strong immunosuppressive properties against TREG cells [181] that contribute to tolerance of malignant tumour cells [182] indicating FTY720 may have potential in post transplant malignancies [183].

### FTY720 inhibits sphingosine kinase 1

**Figure 4:**
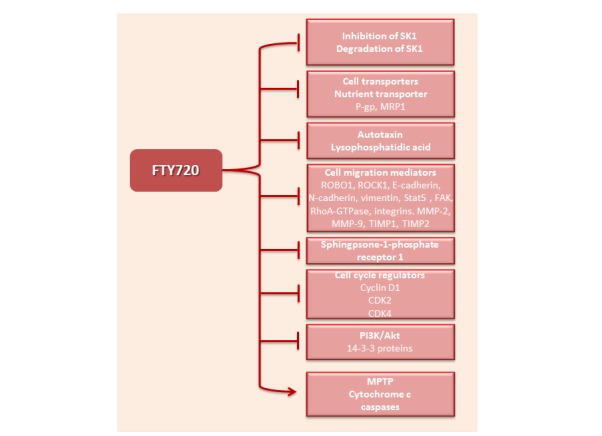
Molecular targets of FTY720 FTY720 inhibits SK1 and blocks the activation of multiple targets of this enzyme. In addition it can directly or indirectly inhibit multiple intracellular targets responsible for cell proliferation, migration and angiogenesis. It further activates mitochondrial permeability transition pore (MPTP), cytochrome c and effector caspases. P-gp - P-glycoprotein, MRP1 - multidrug resistance protein, ROBO1 - roundabout homolog 1, ROCK1 - rho-associated, coiled-coil-containing protein kinase 1, Stat5 - signal transducer and activator of transcription 5, FAK – focal adhesion kinase, MMP – matrix metalloprotease, TIMP – tissue inhibitor of metallopeptidase, CDK - cyclin-dependent kinase.

One of the most studied anticancer mechanisms of FTY720 is inhibition or degradation of SK1 (Figure 4). SK1 downregulation is not dependent on FTY720 phosphorylation; in SK2^−/−^ mice FTY720 decreased SK1 and S1PR1 expression, and eliminated the NFκB/IL-6/STAT3 amplification cascade and development of colitis-associated cancer [184, 185]. FTY720 may inhibit SK1 through multiple mechanisms. In cell lines FTY720 has been shown to inhibit SK1 intracellular activity [88, 108, 122, 123] and it was shown that the *in vitro* IC50 of FTY720 for SK1 is 50uM [108]. FTY720 was demonstrated to be a competitive SK1 inhibitor with respect to sphingosine with an *in situ* Kic of 2 mmol/L [108, 122]. Inhibitor characterization studies reveal that (S)-FTY720 vinylphosphonate inhibits SK1 in an uncompetitive manner, whereas a conjugate of sphingosine with a fluorophore and (S)-FTY720 regioisomer stimulate SK1 activity indicating the presence of allosteric site(s) [122]. Moreover FTY720 and (S)-FTY720 vinylphosphonate, in addition to other direct SK1 inhibitors [186, 187], induce SK1 degradation via ubiquitination and proteasomal processing [108]. This effect could be mediated by accumulation of ceramide and subsequent ceramide-induced activation of the proteasome [131]. In cisplatin-resistant SK-Mel-28 melanoma cells FTY720 induces SK1 degradation by p53-independent caspase activation and may inhibit the PI3K/Akt/mTOR pathway, related to chemoresistance mainly through escape from apoptosis [188]. Conversely in prostate cancer cell lines and mouse tumors FTY720-mediated radiosensitization is facilitated by SK1 inhibition and is caspase independent, suggesting a mechanism involving depletion of prosurvival signaling (e.g., Akt, SK1/S1P) [88]. *In vitro*, SK1 inhibition by FTY720 was shown to lead to prostate cancer cells apoptosis [88] and reduction of the expression of the androgen receptor [134].

### FTY720 reactivates protein phosphatase 2A

Further to its effects on SK1, the non-phosphorylated form of FTY720 has been shown to mediate protein phosphatase 2A (PP2A) reactivation [155, 156]. PP2A is a tumour-suppressor that is down-regulated in many cancers [155, 156, 176, 189-193] resulting in PI3K/Akt/mTOR activation (reviewed in [194]). The FTY720 mediated PP2A reactivation appears important in its apoptosis-inducing effects in many cancers [153, 155, 156, 190, 195, 196]. FTY720 enhanced purified PP2A activity [153] suggesting a direct effect [196], and also induced its reactivation *in vitro* by down-regulation of SET, a PP2A inhibitory protein often upregulated in cancer [155, 156, 176]. Ceramide, structurally similar to FTY720, activates PP2A [197-200], via direct disruption of SET [201]. Sphingosine activates PP2A via disruption of acidic leucine-rich nuclear phosphoprotein-32A (ANP32A) [202]. FTY720 mediated PP2A reactivation induces caspase-dependent apoptosis, affects Akt and extracellular signal-regulated kinase (ERK)-1/2 activation status, and impairs proliferation and clonogenic potential in colorectal cancer cells [176]. In lung cancer cells FTY720 mediated inhibition of SET-driven epithelial-to-mesenchymal transition (EMT), through a SET/PP2A/c-myc/NDRG1/Snail pathway, restored sensitivity to cisplatin, and inhibited invasiveness and growth of lung tumor xenografts [203]. PP2A is deregulated in 59.6% of basal breast tumours and oestrogen receptor negative breast cancer cell lines are sensitive to lower doses of FTY720 [193]. FTY720 mediated activation of the core PP2A complex dephosphorylates the mTOR downstream effectors, 4EBP and S6K, and concurrently releases the block on the p53 pathway [193]. Expression of the PP2A regulatory B subunit B55α (PPP2R2A) is reduced in acute myeloid leukemia cells and suppression of B55α in OCI-AML3 cells induces resistance to FTY-720 [204]. Reactivation of PP2A by FTY720 or its nonimmunosuppressive derivatives (S)-FTY720-OMe, (S)-FTY720-regioisomer and OSU-2S suppressed survival of chronic myeloid leukemia, but not quiescent haematopoietic stem cells *in vitro* and *in vivo* [205]. OSU-2S induces activation of PP2A, phosphorylation at the putative PKC substrate motif, nuclear translocation of SHP1S591 (PTPN6) and deregulation of multiple cellular processes in chronic lymphocytic leukemia (CLL) culminating in potent cytotoxicity [206]. FTY720 disrupted the SET-PP2A interaction, which inhibited BCR-ABL1–recruited JAK2 and impaired β-catenin–dependent survival through GSK-3β activation [205]. In Jak2V617F cells, FTY720 anti-leukaemic activity does not require FTY720 phosphorylation, SET dimerization nor ceramide induction, but depends on interaction with SET K209. Jak2V617F utilizes an alternative SK1-mediated pathway to inhibit PP2A, and FTY720-P acting as a S1P1 agonist elicits signals leading to the Jak2-PI-3Kγ-PKC-SET–mediated PP2A inhibition [207]. Targeting of I2PP2A/SET by FTY720 suppresses lung tumour growth at least in part via PP2A activation and necroptosis mediated by the kinase domain of RIPK1 [208].

### Other targets of FTY720

#### S1PRs

The anti-cancer properties of FTY720 are largely independent of its phosphorylation and effects upon S1P receptors. AAL(S), a close structural analogue of FTY720 that cannot be phosphorylated by SK2, lacks immunosuppressive effects, but induces apoptosis in lymphocytes [209]. Of note, FTY720 induces a downregulation of S1P1 in hepatocellular carcinoma [210]. In Hodgkin lymphoma cells S1P-induced migration was inhibited by an S1PR1 antagonist and FTY720-p, but potentiated by an S1PR2-specific antagonist [211]. In contrast, FTY720-P has been demonstrated to induce growth of breast and colon cancer cells [151].

#### Autotaxin

Autotaxin, an enzyme that produces lysophosphatidic acid from lysophosphatidylcholine in plasma, is associated with increased cancer invasion, metastasis and angiogenesis, and is over-expressed in several human cancers [212-218]. FTY720-p competitively inhibits autotaxin while FTY720 does not [219]. Conversely, FTY720 reduces plasma lysophosphatidic acid in mice [219].

#### Apoptotic pathways

FTY720 induces mitochondrial permeability transition and cytochrome c release via an influence on the permeability transition pore complex and F_0_F_1_-ATPase [159]. Cytochrome c binds to Apaf-1 and activates caspases that induce apoptotic cell death, which is inhibited by Bcl-2 overexpression [159]. Activation of caspases has been implicated in FTY720's pro-apoptotic action [147, 148, 163, 179]. In multiple myeloma cells and rat thymocytes FTY720 induces activation of caspase-8, -9, and -3; poly(ADP-ribose) polymerase cleavage; induces mitochondrial membrane potential and Bax cleavage and translocation of cytochrome c and SMAC/Diablo from mitochondria to the cytosol [157, 220]. FTY720 induces apoptosis of leukemic cells via activation of BIM and BID, which promiscuously bind and inhibit anti-apoptotic Bcl-2 proteins Bcl-2, Bcl- XL and MCL-1, and also activate BAX and BAK [221]. Bcl-2 levels regulate the sensitivity to FTY720 in T cell selective apoptosis [222]. A Fas-independent, Bcl-associated signal transduction pathway and inhibition of ERK activity may be involved in FTY720's anti-cancer properties [223]. The anticancer effect of FTY720 on androgen independent prostate tumour xenografts is mediated through regulating the expression of cell cycle inhibitors such as p21Waf1 and promoting apoptosis through modification of apoptosis regulators such as Bcl-2 and caspases [146].

#### PI3K/Akt

In a liver tumour rat model FTY720 suppresses tumour growth and progression by selective induction of apoptosis of tumour cells via down-regulation of phospho-Akt (ser473) and up-regulation of cleaved caspase-3, together with decrease of focal adhesion kinase [163]. In human prostate cancer cell lines and mouse tumors FTY720-mediated radiosensitization is caspase independent and linked to SK1 inhibition and downregulation of p-Akt [88]. In human breast cancer cells FTY720 potentiates radiation effects through perturbation of PI3K/Akt and p42/44 mitogen-activated protein kinase MAPK [224]. FTY720 down-regulates IL-6-induced phosphorylation of Akt, signal transducers and activators of transcription 3 (Stat3), and MAPK; insulin-like growth factor-I-triggered Akt phosphorylation; and TNFα-induced Iκα and NFκB p65 phosphorylation [157]. In neuroblastoma cells FTY720-induced cell death, alone or in combination with topotecan, is caspase-independent and induces dephosphorylation of Akt and its downstream effector BAD with release of cytochrome c, which the authors suggested to be due to involvement of 14-3-3 proteins [225]. Indeed, FTY720 and sphingosine bind directly to and regulate the function of pro-survival ubiquitous phospho-serine binding 14-3-3 proteins. Expression of non-phosphorylatable 14-3-3 in cells attenuates apoptosis upon FTY720 treatment [226] and protein kinase A [227] and PKCsigma [228] phosphorylate 14-3-3 in a sphingosine-dependent manner. Recently it was suggested that FTY720 induced inhibition of PI3K/Akt pathway is mediated by phosphorylation of PP2A [229].

FTY720-induced inhibition of PI3K/Akt pathway downregulated mTOR signalling, which was shown to be crucial for FTY720-mediated inhibition of migration and invasion of glioblastoma cells [230]. mTOR is a key player in prosurvival cell signalling most notably regulating transcription and activity of multiple signalling molecules through its downstream targets S6 kinase and EIF4E transcription factor. It was demonstrated that FTY720-induced chemo-sensitization of cisplatin resistant melanoma cells is mediated by reduction of mTOR activity and the decrease in epidermal growth factor receptor expression [188].

#### Cell cycle

FTY720 treatment results in time-dependent downregulation of cyclin D1 and accumulation of cells in G(0)-G(1) and G(2)-M phases of the cell cycle with concomitant decrease in S-phase entry [154]. In prostate cancer cells FTY720 acts through modulation of mitogenic signaling, cell-cycle regulators (e.g. a decrease in CDK2 and CDK4 and induction of Cip1/p21) and induction of G_1_ arrest, and apoptotic death mediated by mitochondrial death pathway as well as the contribution of FAK to MAPK pathways [147].

#### Cell transporters

Some report propose that FTY720's anti-cancer activity may be due to its ability to induce nutrient transporter down-regulation [231] or inhibition of P-glycoprotein (P-gp) and multidrug resistance protein [174].

#### Autophagy

The evidence about the role of FTY720 in autophagy is controversial. FTY720 can induce U266 multiple myeloma cell apoptosis and autophagy with reactive oxygen species (ROS) regulating both of these processes [232]. However, this is not always beneficial, since in a variety of ovarian cancer cell lines including cisplatin-sensitive and cisplatin-resistant cells, the autophagy induction by FTY720 was antagonistic to cisplatin-mediated apoptosis [233]. A recent study shows that a combination of FTY720 and γ-irradiation blocks the autophagy flux causing a paradoxical increase of autophagosomes in breast cancer cells that die through apoptosis [224]. Finally, it was reported that FTY720 was effective in limiting murine metastatic melanoma development *in vivo* and induced apoptosis regulated by ROS and by increased expression of β-catenin *in vitro* without indications of autophagy or necroptosis [234].

#### Cell migration

FTY720 may reduce cell invasion and migration through several mechanisms. FTY720 modulates roundabout homolog 1 (ROBO1), rho-associated, coiled-coil-containing protein kinase 1 (ROCK1) and epithelial to mesemchymal (EMT) related factors such as E-cadherin, N-cadherin and vimentin [235]. FTY720 down regulates matrix metalloprotease (MMP)-2 & MMP-9 and upregulates tissue inhibitors of metalloproteinases: TIMP1 & TIMP2 [236]. Finally, FTY720-mediated reduction ion cell migration was reported to be mediated by its effects on Bcl-2 [146, 154, 167, 170], Stat5 [156], PI3K/Akt/mTOR/p70S6K pathway [147, 154, 236, 237], FAK [147], RhoA-GTPase [149] and integrins [167].

## EXPERT COMMENTARY

The fact that FTY720 is an FDA-approved drug for treatment of progressive multiple sclerosis [238, 239] can significantly simplify its clinical implementation for other uses, in the case that a clinical benefit is demonstrated. However, despite its promising actions against a diversity of cancers, FTY720's S1PR-mediated immunosuppressive effects involving T-cell sequestration to lymph nodes limit its potential in cancer treatment. T-cells are considered as one of the most important mechanisms of anti-cancer defence and phosphorylated FTY720 inhibited random migration, cytotoxicity and tumour infiltration of activated CD3(+)NKG2D(+)CD8(+) T-lymphocytes in a mouse xenograft model [240]. In addition to its direct antitumour effect, FTY720 has strong immunosuppressive properties, specifically against regulatory T cells [181], which can contribute to tolerance of malignant tumour cells [182]. It has therefore been suggested to evaluate the use of FTY720 in patients with post-transplant malignancies [183]. There are several reports suggesting a direct influence of FTY720-p on cancer cells ranging from induction of growth in breast and colon cancer cells to inhibition of cancer cell migration. Currently, there is no consensus about the overall role of FTY720-p in cancer progression.

These potentially undesired effects of FTY720-p can be overcome by several ways. One potential way is by blocking FTY720 phosphorylation. OSU-2S, a synthetic derivative of FTY720, demonstrates more potent anti-tumour activity and lacks S1PR-mediated immunosuppressive effects [241]. OSU-2S displays satisfactory pharmacokinetic properties as shown using a liquid chromatography-tandem mass spectrometry (LC-MS/MS) [242]. Through CCL tumour antigen ROR1-targeted delivery, OSU-2S induces activation of PP2A, phosphorylation and nuclear translocation of SHP1S591 and deregulation of multiple cellular processes in CCL resulting in potent cytotoxicity [206].

Another way of limiting the immune suppressing effects of FTY720 is its tissue targeting and release control. A liposomal carrier of FTY720 (LP-FTY720) exhibits high drug loading ratio, prolonged *in vitro* release rate and beneficial pharmacokinetic properties *in vivo* compared to free FTY720 [243]. Incorporating tumour specific antibodies (anti-CD19, anti-CD20 and anti-CD37) achieved higher delivery and killing efficiency in primary CLL cells *ex vivo* which may be beneficial for targeting hematologic diseases where FTY720 induces T cell apoptosis [243]. Enhanced targeting of FTY720 through CD37 and CD19 dual immunoliposomes may improve the clinical efficacy of FTY720 in B-Cell lymphocytic leukaemia [244].

Alternatively, the immunosuppressive action of FTY720 can be reduced by a blockade of immune inhibitory pathways using anti-CTLA-4 mAb, anti-PD-L1 mAb, and/or the indoleamine-pyrrole 2,3-dioxygenase (IDO) inhibitor INCB23843 which restored IL-2 production, proliferation of intratumoural T cells, and tumour growth control in FTY720 treated murine B16.SIY melanoma model [245].

Another way of exploiting FTY720's anti-cancer activity is to mimic its effects on downstream targets using alternative small molecule inhibitors. An example of such approach is FTY720-induced nutrient transporter down-regulation [231]. O-substituted benzyl ethers of pyrrolidines induce nutrient transporter down-regulation and lack FTY720's S1P receptor-related dose-limiting toxicity in human leukaemia cells [231].

Importantly, in many of the cancer studies cited in this review, FTY720 was applied at a dosage in excess of that used in multiple sclerosis patients, who currently receive 0.5 mg once-daily dose. The known adverse effects at this dose include: lymphopenia, increased alanine aminotransferase, herpes zoster infection, hypertension, first-dose bradycardia, and first-degree atrioventricular block [239, 246], reviewed in [88]. Higher doses of FTY720 that may be necessary for cancer treatment may be associated with more adverse events or unpredictable off-target effects, and this needs to be addressed by further studies.

At the moment it is unlikely that FTY720 may be used as a monotherapy for any cancer, at least in its pure form. However, a multitude of studies has shown its potentiating effect on many therapies including standard DNA-targeting and antimitotic therapies and γ-irradiation. Therefore, one of the most important steps in its clinical implementation is finding the key combinations where FTY720 can act in synergy with the currently used therapies inducing sensitisation and overcoming cancer therapy resistance.

Another important step is defining the patient populations that will most benefit from the FTY720 therapy. This could be largely based on the tumour expression of the multiple FTY720 targets described in this review. It may be that some subsets of tumours would be particularly sensitive to this therapy. This approach can be helped by the large scale sequencing studies currently undertaken in several countries with the aim of defining specific cancer mutations/genetic aberrations in large groups of patients. For example it was shown that cancer cells overexpressing pp32r1 or a pp32r1Y140H functional mutant in the ANP32C oncogene that is overexpressed in breast, prostate and pancreatic tumours, may demonstrate enhanced resistance to FTY720 treatment through conserved residue F136, likely to be a key determinant of the FTY720 binding site [247]. However it is not known whether this mutation is present in large human populations.

Overall, FTY720 is a clinically approved therapy for multiple sclerosis and a potent apoptosis inducer and anticancer agent with a proven efficiency in multiple *in vitro* and *in vivo* anticancer models. While the clinical transition of FTY720 is currently limited by its immune suppression effects, in our opinion studies aiming at the FTY720 delivery and release together with identifying its key synergetic combinations and relevant patient subsets may lead to its re-evaluation and rapid introduction into the clinic.

## FIVE-YEAR VIEW

We hypothesise that in 5 years the use of targeted FTY720 delivery or its specific non-immunosuppressive analogues will allow its clinical trials for treatment of cancer. Its combinations with other chemotherapies may prove more efficient than its use as a monotherapy.

## KEY ISSUES

FTY720 demonstrates a proven efficacy in multiple *in vitro* and *in vivo* cancer models.

FTY720 inhibits sphingosine kinase 1, a proto-oncogenic enzyme with *in vitro* and clinical cancer association.

FTY720's has actions on several other molecular targets including protein phosphatase 2A, the PI3K/Akt pathway, cell cycle regulators, cell transporters, autotaxin and the mitochondrial permeability transition pore.

FTY720 is a FDA-approved drug for multiple sclerosis, which can significantly simplify its clinical implementation for other uses.

Targeted FTY720 delivery and release together with identifying its key synergetic combinations and relevant patient subsets may lead to its rapid introduction into the clinic.
